# Pre- or Perioperative Immunotherapy Combined with Chemotherapy Versus Chemotherapy Alone in Resectable Non-Small Cell Lung Cancer (NSCLC): A Systematic Literature Review

**DOI:** 10.3390/cancers18122002

**Published:** 2026-06-20

**Authors:** Sophie Lehner, Josef Singer, Klaus Hackner, Karin Armster, Wolfgang Dietl, Bahil Ghanim

**Affiliations:** 1 Karl Landsteiner University of Health Sciences, 3500 Krems, Austria; 2 Department of Internal Medicine 2, Karl Landsteiner University, University Hospital Krems, 3500 Krems, Austria; 3 Department of Pneumology, Karl Landsteiner University, University Hospital Krems, 3500 Krems, Austria; 4 Department of Cardiothoracic Surgery, Clinic Oberwart, 7400 Oberwart, Austria

**Keywords:** NSCLC, immunotherapy, immune checkpoint inhibitors, chemoimmunotherapy, survival, pathological response, adverse events, systematic review

## Abstract

**Background/Objectives**: Immunotherapy has emerged as an important field of research in non-small-cell lung cancer (NSCLC) and has demonstrated promising results in clinical practice. In recent years, multiple studies have been conducted, increasing the amount of available data. Therefore, the aim of this systematic review is to assess the combination of perioperative immunotherapy with chemotherapy compared to chemotherapy only in patients with resectable NSCLC in terms of survival, pathological response, and adverse events. **Methods**: The clinical databases PubMed, Cochrane Library, ClinicalTrials.gov, and the World Health Organization International Clinical Trials Registry Platform (WHO ICTRP) were systematically searched, up to March 2026. A two-step selection process served as the screening for eligibility, in which the assessment was based on pre-defined inclusion and exclusion criteria. This process was visualized via a PRISMA diagram. For each included study, the risk of bias was assessed with the help of the Cochrane Risk of Bias 2.0 tool and the Newcastle Ottawa Scale. A narrative synthesis was performed due to heterogeneity. Data were extracted into tables. **Results**: A total of 16 studies, involving 4646 patients in total, met the eligibility criteria, and their data on study population, intervention, comparison, and outcome were extracted into tabular form. Survival and pathological response rates are continuously higher in patients treated with immunochemotherapy. Findings on adverse events differed across the individual studies, though the results indicate an increased risk of treatment-related adverse events (TRAEs) in patients undergoing the combined treatment approach. **Discussion/Conclusions**: Chemoimmunotherapy leads to superior clinical outcomes in terms of survival and pathological response rates, though the trend towards a higher incidence and severity of TRAEs warrants further research. The interpretation of findings is limited by differences in study characteristics, mechanism of conduct, and endpoints between the individual studies.

## Simple Summary

Adding immunotherapy to a (pre- or perioperative) chemotherapeutic regimen has been a game changer in patients with resectable non-small-cell lung cancer (NSCLC). Findings of previous studies attributed a substantial survival benefit to this combined treatment approach. Results on adverse events and safety varied. Additional studies continue to be released, increasing the existing pool of evidence. Therefore, this systematic review aims to compare survival, pathological response, and adverse events in NSCLC patients treated with immunochemotherapy versus chemotherapy alone. The combination of immunotherapy with chemotherapy in patients with resectable NSCLC yielded superior survival outcomes and higher pathological response rates in all included studies. Findings on adverse events were heterogeneous, and the extent of data reporting varied across the individual studies. However, there is a trend towards an increased frequency and severity of adverse events in patients treated with immunochemotherapy, highlighting the importance of patient selection and the need for further research.

## 1. Introduction

Lung cancer is still the most commonly diagnosed malignant neoplasm worldwide and continues to cause the highest number of cancer-related deaths [1]. With approximately 85%, non-small-cell lung cancer (NSCLC) is the most frequent subtype [2]. Depending on histopathological characteristics, NSCLC can be divided into three major categories: adenocarcinoma, squamous cell carcinoma, and large cell carcinoma [3,4].

Within the last decade, immunotherapy with immune checkpoint inhibitors has been established as an important treatment option for NSCLC patients. Several studies reported its clinical efficacy [5,6], especially in NSCLC patients without targetable genetic mutations [7,8].

Malignant cells can escape immune surveillance by expressing checkpoint molecules on their surface, with programmed death-ligand 1 (PD-L1) being among the most frequently expressed checkpoint molecules. When the programmed cell death protein 1 (PD-1) is bound by the PD-L1 on the tumor cell, the action of T-cell-associated kinases is blocked, and thus the T-cell-mediated destruction of the respective tumor cell is inhibited. Furthermore, the release of B-cell proliferative cytokines such as Interleukin-2 is also blocked by the stimulation of the PD-1, additionally suppressing the specific immune response against the malignant cells. Immune checkpoint inhibitors such as Nivolumab, Pembrolizumab, and Tislelizumab are monoclonal antibodies targeting the PD-1 receptor on the T-cell. On the other hand, Atezolizumab or Durvalumab target the PD-L1 on the tumor cell, thereby preventing the PD-L1-mediated negative stimulation of PD-1. An additional way to inhibit immune checkpoints are the cytotoxic T-lymphocyte-associated protein 4 inhibitors, such as Ipilimumab [8].

Surgery still plays an important role in the standard of care for both early and late-stage NSCLC as the treatment of choice or part of a multi-modality approach, respectively [4].

Chemotherapy remains an integral part of NSCLC treatment across all disease stages, yet its use has evolved from a standalone therapy to a combined treatment strategy [7].

Over the past decade, several clinical studies attributed improved outcomes for NSCLC patients treated with neoadjuvant or perioperative immunochemotherapy compared with patients undergoing chemotherapy alone. The most common primary endpoints of interest of the respective studies are survival and pathological response rates. The differences in adverse events and safety between the intervention and comparison groups are often evaluated as well, though findings vary across the individual studies [5,6]. Literature reviews focusing on immunochemotherapy in patients with resectable NSCLC in terms of survival, pathological response, and safety are available, though their number remains limited. Moreover, as there is a continuous increase in newly published primary studies, the amount of data available is rising, highlighting the need for further systematic reviews [5,8,9].

Therefore, the aim of this systematic review is to analyze current findings on survival parameters (i.e., overall survival (OS), progression-free survival (PFS), disease-free survival (DFS), and event-free survival (EFS)), pathological response (pathological complete response (pCR), major pathological response (MPR)), and side effects in patients with resectable NSCLC treated with neoadjuvant or perioperative immunochemotherapy, and to compare their outcomes to patients who received standard chemotherapy without immunotherapy.

## 2. Materials and Methods

### 2.1. Eligibility Criteria

Studies were eligible if their population group was limited to adult patients with stage IB-IIIB resectable NSCLC. Their intervention groups received neoadjuvant or perioperative immunotherapy (immune checkpoint inhibitors) in combination with platinum-based chemotherapy, while the comparison arm was treated with neoadjuvant or perioperative platinum-based chemotherapy alone. Studies including patients with unresectable or metastatic disease as well as participants with known epidermal growth factor receptor (*EGFR*) or anaplastic lymphoma kinase (*ALK*) mutations were excluded. Eligible studies for this review assessed survival outcomes (OS, PFS, DFS, EFS), pathological response rates (pCR, MPR), and/or safety and adverse events. The included studies of this review were either randomized controlled trials or prospective/retrospective cohort studies. While the literature search included systematic reviews also, the use of studies of this design was limited for background information and discussion. The search was limited to studies in English and German that were published from 2015 onward due to the emergence and clinical integration of immunotherapy.

### 2.2. Information Sources and Search Strategy

A systematic literature search (last updated in March 2026) was conducted in the clinical databases PubMed, Cochrane Library, ClinicalTrials.gov, and the World Health Organization International Clinical Trials Registry Platform (WHO ICTRP). The search was based on the pre-defined inclusion and exclusion criteria of this review. For representation, the search in PubMed/MEDLINE is provided in Appendix A (Table A1).

### 2.3. Study Selection

EndNote ^®^ (Version EndNote 21.5) [10] was used as the reference management software for this review. Groups and subgroups in EndNote served as a tool for organization and subsequent classification throughout the selection process. After duplicate removal, the screening for eligibility was performed in a two-step process: first, the title and abstract of all studies were screened. Subsequently, the full texts were assessed, resulting in the exclusion of ten sources. As subgroup analyses and secondary publications exist for many of the included studies, the number of reports per study is high. The entire selection process was guided by the inclusion and exclusion criteria of this review and performed by SL and BG.

### 2.4. Data Extraction

The key study and population characteristics including author, year of publication, country of conduct, study design, sample size, disease stage, age, and sex of participants were extracted in the form of a table. In a next step, data on intervention and comparison characteristics were extracted in the same way, containing information about the type(s) and timing of immunotherapy applied in the intervention arm and type(s) of chemotherapy applied in the intervention and comparison arms. Finally, outcomes on survival, pathological response (pCR, MPR), and adverse events (TRAEs any grade, grade ≥ 3 TRAEs, serious TRAEs, discontinuation due to TRAEs, fatal TRAEs) were extracted into three individual tables. While most studies explicitly stated their use of the graded severity scale (grade 1–5) for assessing treatment-related adverse events (TRAEs) based on the Common Terminology Criteria for Adverse Events [11], some did not provide specific information about the type of system applied. For data on survival and pathological response, the corresponding effect measures were extracted in the form of hazard ratio, confidence interval, and *p*-value. The process of data extraction was performed independently by SL and BG.

### 2.5. Risk of Bias Assessment

The Cochrane Risk of Bias 2.0 tool [12,13] and the Newcastle Ottawa Scale [14,15] were used to assess the risk of bias in randomized controlled trials (RCTs) and prospective/retrospective cohort studies, respectively. The assessment was conducted independently by SL and BG.

### 2.6. Data Synthesis

The evidence synthesis was done in a narrative way due to heterogeneity in study design, outcome measures, and data reporting. The individual studies differed in the type(s) of immunotherapeutic and chemotherapeutic agents applied, the corresponding treatment schedule, endpoints of interest, and follow-up duration. This systematic review was conducted in accordance with the PRISMA 2020 guidelines. Further information is provided via the PRISMA 2020 Flow Diagram of Study Selection and the PRISMA 2020 Checklist in the Supplementary Materials Tables S1 and S2 [16]. The study protocol of this review was not registered.

### 2.7. Ethical Consideration

As the information used for this review did not include any personal data, no ethical approval was required.

### 2.8. Reporting Bias Assessment

As no quantitative meta-analysis was conducted, the risk of reporting bias could not statistically be assessed.

### 2.9. Certainty of Evidence

The certainty of evidence was put into context by highlighting possible limitations such as inconsistency and heterogeneity.

## 3. Results

### 3.1. Selection Process

**Figure 1:**
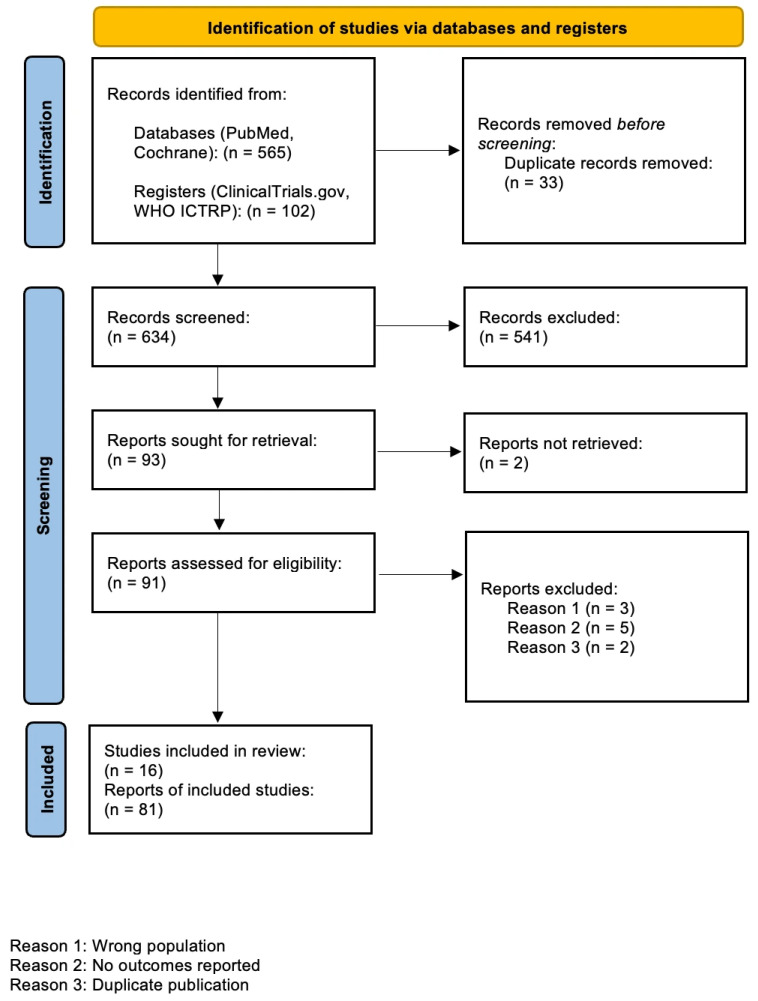
PRISMA 2020 flow diagram of study selection, adapted from Page et al. [16].

After duplicate removal, the title and abstract of 634 studies were screened for eligibility. The full texts of the remaining 91 studies were assessed in the second step of the selection process. This led to a total of 16 eligible studies for this systematic review. The PRISMA flow diagram [16] (Figure 1) provides an overview of this process and explains reasoning in the case of exclusion.

### 3.2. Risk of Bias

The risk of bias of the included studies ranged from low to moderate risk. In the case of prospective and retrospective cohort studies, their susceptibility to selection bias and confounding should be taken into consideration. The detailed risk of bias assessment is depicted in Table 1 and Table 2 [12,13,14,15].

### 3.3. Study and Population Characteristics

The included studies were published between 2018 and 2025. The number of RCTs and cohort studies was equal. Three out of the eight RCTs were conducted in an open-label design while the remaining five had a double-blinded design. The majority of studies were conducted in a single country, and the study design included single- as well as multicenter studies. The prominent CheckMate 77T [19], KEYNOTE-671 [6], AEGEAN [20], and CheckMate 816 study [5] were examples of RCTs that involved multiple nationalities. Sample sizes were heterogeneous and ranged from 55 to 802 patients. Furthermore, group allocation differed across the studies. Five studies distributed the participants equally among the intervention and comparison arm. The remaining 11 studies reported imbalances between approximately 0.2% to 52.7%.

Three publications analyzed patients with disease stages that did not meet the eligibility criteria of this review. However, as the proportion of patients who did not meet the inclusion criteria was small, the information of these publications is contained in the evidence synthesis. The respective studies and number of patients missing inclusion criteria are as follows:

Wang et al. (2025) [25] included one patient with stage IIIC NSCLC (in the comparison group).

The NeoR-World study [27] also covered stage IA patients. However, the percentage of patients with stage I in both intervention and comparison arms (including patients with oncogenic mutations) was only 8.6% and 9.8%, respectively.

Yang et al. (2018) [30] reported four patients with stage IV (in the comparison group, making up 10% of the comparison arm and approximately 7.27% of the total sample size).

Patients with known *EGFR* and *ALK* mutations were included in the NeoR-World study [27], as mentioned before, but, as their analyses could be isolated from patients without oncogenic mutations, the respective information was used.

The median age among all studies was comparable between the intervention and control group and ranged from 52.5 to 70 in the intervention group and from 50.5 to 70 in the comparison group. Male sex was most prominent in the majority of all studies.

Table 3 provides a detailed overview of study and population characteristics of the individual studies.

### 3.4. Intervention and Comparison Characteristics

The studies differed in type and number of immunotherapeutic agents; nine out of the 16 studies limited the application of immunotherapy to a single agent while the other five included a range between four to seven different types. Two studies did not provide explicit information on the type of immunotherapeutic agent used. While seven publications applied immunotherapy in a neoadjuvant setting only, patients in the intervention groups of the other nine studies were treated with perioperative immunotherapy.

Further information on intervention and comparison characteristics is depicted in Table 4.

### 3.5. Outcome Characteristics

#### 3.5.1. Survival Outcomes

All studies reported superior survival outcomes in patients receiving immunochemotherapy compared to patients in the control group. The statistical significance of the respective data differed, and 10 results did not include information about statistical significance. The median DFS, EFS, or OS of the intervention arm were not yet reached in some studies, implying that less than 50% of patients in this group had experienced a relapse, an event, or died at the time of evaluation.

A detailed overview of the survival outcomes is provided in Table 5.

#### 3.5.2. Pathological Response Outcomes

Outcomes of pCR were assessed by all 16 studies while MPR results were reported by 14 of them. Each study attributed higher rates of pCR to the intervention arm, and the same applied for the MPR rates reported by the 14 studies. Most publications included the data’s statistical significance and, among those, two outcomes of pCR were statistically not significant. The difference in pCR between intervention and comparison groups varied from 3 to 35.0 percentage points. Patients in the intervention group had a MPR of 19.2 to 50.2 percentage points higher than the comparison group. The respective findings of Liu et al. [29] were considered separately as the study reported a combination of pCR and MPR.

Detailed information on each study’s pathological response outcomes is presented in Table 6.

#### 3.5.3. Adverse Events and Safety Outcomes

Findings on TRAEs were reported by most studies though direct comparability is limited by the heterogeneous reporting of the respective studies. The occurrence of any kind of TRAEs was an endpoint of most studies, with the respective minimum being 80.4% in the intervention arm and 63.6% in the comparison arm. According to eight of those studies, a higher frequency of any kind of TRAE was observable in patients receiving chemoimmunotherapy, while three studies showed increased rates in patients undergoing chemotherapy only. There was a relative difference of up to 21 percentage points between the two arms. The occurrence of grade ≥ 3 TRAEs ranged from 4.6% to 73% between the individual intervention groups and from 8% to 67.3% between the comparison groups. Nine studies attributed higher rates of grade ≥ 3 TRAEs with the addition of immunotherapy, while three reported a higher incidence in the chemotherapy-only arm. The relative difference varied between 0.5 and 28.7 percentage points. A total of five studies assessed the incidence of serious TRAEs; respective data of each of them showed higher rates in patients undergoing the combined treatment approach. This was also the case for the seven studies providing data on TRAEs, leading to discontinuation where chemoimmunotherapy consistently led to a higher incidence of this TRAE. The relative difference between the two arms varied from 1.7 to 9.7 and 0.5 to 11.9 percentage points, respectively. Outcomes on TRAEs resulting in death were reported by eight studies: five of them observed a higher rate in the intervention arm, while one study attributed a higher rate to the comparison arm. No difference between the two groups was found in two of the eight studies.

In the RATIONALE-315 study [17,31], half of the fatal TRAEs in the intervention arm (i.e., two out of the four fatal TRAEs) were specifically linked to an immune-mediated cause. One out of the four fatal TRAEs in the intervention group of the KEYNOTE-671 study [6,33] was explicitly found to be immune-mediated. In the AEGEAN study [20], seven fatal TRAEs occurred in the intervention group, yet only one was attributed to an immune-mediated origin. In the remaining studies reporting cases of fatal TRAEs in the immunotherapy arm, no specific information on immunotherapy-induced fatality was provided.

Generally, the TRAEs ranged from fatigue and gastrointestinal disturbances to hematological complications such as anemia and neutropenia, neuropathy, immune-mediated pneumonitis, and death [6,20,21].

Table 7 provides a detailed overview of adverse events outcomes.

#### 3.5.4. Other Safety-Related Outcomes

Some studies reported specific endpoints beyond the scale of Table 5, Table 6 and Table 7:

The retrospective cohort study of Lei et al. (2025) [23] exclusively enrolled participants with a confirmed pulmonary lymphoepithelioma-like carcinoma (PLELC) diagnosis. Patients receiving chemoimmunotherapy experienced superior survival outcomes as well as higher pathological response rates, though the occurrence of TRAEs was slightly increased in this group. Detailed data reports are provided in Table 5, Table 6 and Table 7. Additionally, based on their findings, Lei et al. (2025) [23] suggested that patients with a PLELC diagnosis may respond more favorably to chemoimmunotherapy compared to other NSCLC patients.

The study of Shuai et al. [24] focused on elderly patients (≥65 years) and assessed the effectiveness of neoadjuvant immunotherapy combined with chemotherapy in terms of survival, pathological response, and TRAEs. Participants in the intervention group of this retrospective cohort study experienced both higher survival as well as pCR and MPR rates. However, a higher occurrence of ≥3 TRAEs was also observed in this treatment group, yet no new or unexpected side effects in the respective study population occurred. Data associated with these results are depicted in Table 5, Table 6 and Table 7.

The aim of Wang et al. [26] was to assess perioperative opioid (sufentanil, remifentanil, morphine) use following the addition of immunotherapy, and to compare it with chemotherapy-only. For statistical comparison, the doses of remifentanil and morphine were converted into a sufentanil-equivalent dose with a conversion ratio of remifentanil 100 μg = morphine 10 mg = sufentanil 10 μg. The findings of this prospective cohort study indicate a higher need for opioids in patients receiving the combined treatment regimen, as these patients required opioid analgesics with a mean of 334.4 μg compared to 274.0 μg in the comparison arm (*p* < 0.001).

## 4. Discussion

Treating NSCLC patients in a neoadjuvant or perioperative setting with both chemotherapy and immunotherapy leads to improved outcomes in terms of survival and pathological response rates compared to those undergoing chemotherapy only. These findings are reported by all included studies, strengthening the confidence in this evidence.

These superior outcomes associated with chemoimmunotherapy could be explained by the synergistic effect: beyond inducing tumor cell death, chemotherapy also increases immunotherapeutic efficacy by priming the immune system. This challenges the longstanding classification of chemotherapy as an immunosuppressive agent. In fact, under certain conditions, chemotherapy modulates the patient’s immune system through a variety of mechanisms, such as targeting immunosuppressive cells, inducing immunogenic cell death (ICD), thereby stimulating a T-cell response, activating natural killer (NK) cells, as well as increasing antigen presentation and immune cell infiltration. Moreover, different chemotherapeutic agents exhibit different immunomodulatory effects: unlike many other platinum compounds, Cisplatin is not considered a true inducer of ICD. However, it stimulates the immune system by targeting immunosuppressive cells, modulating antigen presentation and immune cell infiltration. Other platinum-based agents and taxanes, on the other hand, are established inducers of ICD, thereby potentially enhancing the efficacy of PD-1/PD-L1 inhibition. Gemcitabine, for example, has been shown to be highly effective at NK cell activation when administered at low doses. Moreover, the immunomodulatory capability not only varies between different chemotherapeutic agents, but is also influenced by the dosage and timing of the respective agent. However, further research is needed to determine optimal treatment schedules [35,36].

A general trend towards a higher incidence and severity of TRAEs with the application of immunotherapy can be observed, even though inconsistent reporting, variable number and extent of endpoints, as well as heterogeneous results limit comparability. A possible explanation for the inconsistent reporting on TRAEs might be the primary focus of most studies on survival and pathological response outcomes. The occurrence of adverse events was classified and reported as any TRAEs, grade ≥ 3 TRAEs, serious TRAEs, discontinuation due to TRAEs, and fatal TRAEs. While the data on any TRAEs, grade ≥ 3 TRAEs, and fatal TRAEs are characterized by heterogeneity, the occurrence of severe TRAEs and discontinuation due to TRAEs is consistently higher in patients undergoing chemoimmunotherapy. However, it should be noted that in the intervention groups, TRAEs were assessed for the combination of immunotherapy with chemotherapy. In the case of fatal TRAEs in the intervention arms, 14% to 50% of fatalities were explicitly linked to an immune-mediated cause.

Outcomes of grade ≥ 3 TRAEs differ most notably across the individual studies.

Chemoimmunotherapy was linked with higher rates by the grade ≥ 3 TRAEs. While, for example, RATIONALE-315 [17] and Neotorch [18] reported higher TRAEs in patients treated by chemoimmunotherapy, data of the CheckMate816 study [5] showed lower rates of these adverse events in the intervention group.

A notable anomaly is observable in the findings of Zhou et al. [28]: the study reported significantly lower rates of severe TRAEs in the intervention arm (4.6% vs. 33.3%), which is discordant with the findings of RATIONALE-315 [17], attributing a rate of 73% to patients receiving chemoimmunotherapy. This deviation might be due to the small sample size of Zhou et al. [28], with a total of only 59 analyzed patients.

However, the heterogeneity in TRAEs remains when limiting the comparison to studies with larger sample sizes (>350). This is also the case when comparing RCTs only. The comparison between type and number of immunotherapeutic agents in terms of incidence and severity of adverse events leads to an observable trend: the highest rates of grade ≥ 3 TRAEs occurred in patients receiving the PD-1 inhibitors Tislelizumab [17] and Toripalimab [18]. The rates of grade ≥ 3 TRAEs associated with the application of Nivolumab were heterogenous across the respective studies, although there is a trend indicating lower rates compared with other agents [5,19,21,24,27,28,29]. A higher incidence of any TRAEs and grade ≥ 3 TRAEs is observable in studies focusing on a single immunotherapeutic agent only [5,6,17,18,19,20,21,22,23,30]. Studies based on a multi-agent approach tend to have a lower rate of grade ≥ 3 TRAEs [23,24,27,28,29]. Moreover, these interpretations are limited by the lack of comparative analyses, and should therefore be interpreted cautiously. Further research comparing toxicities between the respective groups is required to allow for confident conclusions.

Moreover, TRAEs in the intervention groups were reported for the combined therapy, and only some studies provided further information on the proportion of immune-mediated adverse events. In fact, data of the CheckMate 816 trial [5,34] indicated that while the addition of immunotherapy slightly increased immune-mediated adverse events, it did not put the intervention group at a higher risk of experiencing other adverse events. In fact, the overall incidence of TRAEs was lower in patients treated with immunochemotherapy. In the KEYNOTE-671 study [6,33], patients in the intervention group were also more susceptible to immune-mediated adverse events, and the data also show a slightly higher total rate of TRAEs in this group. This was also the case for the AEGEAN study [20,37], in which the intervention arm was more prone to immune-mediated adverse events, though rates of general adverse events were not significantly higher in patients treated with immunochemotherapy. In fact, the occurrence of grade ≥ 3 TRAEs was slightly lower in the intervention group.

When comparing safety and adverse events in terms of surgical success, the addition of immunotherapy does not lead to increased surgical risks, and in fact might increase surgical success in terms of complete tumor clearance with negative margins, according to an updated version of AEGEAN [37]. In fact, according to the CheckMate 816 investigators [5,34], patients in the immunochemotherapy group achieved more favorable surgical outcomes across several outcome measures. Patients in the intervention arm were more likely to undergo definite surgery (83.2% vs. 75.4%) and to be eligible for a minimally invasive approach (29.5% vs. 21.5%), with the rate of conversion to thoracotomy after starting with minimally invasive surgery being lower in this group (11.4% vs. 15.6%). Furthermore, the need for a pneumonectomy was lower in patients treated with Nivolumab compared to the control group (16.8% vs. 25.2%). Moreover, the success of completing tumor resection was higher in the intervention arm, with higher R0 rates (83.2% vs. 77.8%) and lower R1 rates (10.7% vs. 15.6%). R2 rates were similar between the two groups (3.4% vs. 3.0%) Also, the duration of surgery was notably shorter in the intervention arm, with a median of 185.0 min compared to a median of 213.5 min in the comparison group. However, despite the differences in surgical approach rates, the median length of hospital stay was identical for both arms with 10.0 days. Nevertheless, the incidence of surgery-related adverse events was lower in the immunochemotherapy group for both any grade (41.6% vs. 46.7%) and grade 3 or 4 (11.4% vs. 14.8%).

A common concern regarding neoadjuvant immunotherapy is that patients might not respond and potentially lose eligibility for curative surgery due to disease progression. However, data of the CheckMate 816 trial [5,34] demonstrate the opposite: not only did more patients in the intervention group undergo definite surgery, but also fewer cancelations of surgery occurred (15.6% vs. 20.7%). In fact, cancelations due to disease progression were recorded in 6.7% in the intervention arm compared to 9.5% in the comparison arm. In the KEYNOTE-671 study [6,33], findings are similar, with more patients in the intervention group undergoing surgery than in the comparison group (82.1% vs. 79.4%). Also, patients treated with immunochemotherapy were less likely to have their surgery canceled (17.9% vs. 20.5%), and cancelation rates caused by disease progression were also lower in the intervention arm (4.1% vs. 8.2%). In the AEGEAN trial [20,37], a slightly lower percentage of patients in the intervention arm underwent surgery (81.0% vs. 81.3%) and the overall rate of surgery cancelation due to any cause was slightly higher in patients treated with immunochemotherapy (19.0% vs. 18.7%). However, cancelations due to disease progression were slightly less likely in the intervention group than in the comparison group (6.8% vs. 7.5%). These findings highlight the need for a predictive biomarker to reduce the number of patients progressing beyond surgical resectability due to failed neoadjuvant therapy.

PD-L1 expression levels are commonly used as an indicator for responsiveness. The RCTs included in this review determined PD-L1 expression using the VENTANA PD-L1 (SP263), PD-L1 IHC 28-8 pharmDx, or the PD-L1 IHC 22C3 pharmDx assays [5,6,17,18,19,20,21,22,31,32,33,34]. However, PD-L1 expression is not a reliable parameter to determine eligibility. In fact, the CheckMate 816 study [5,34] demonstrated that patients across all PD-L1 expression levels had beneficial outcomes. However, higher PD-L1 expression levels correlated with higher outcome benefits. Therefore, determining a patient’s eligibility for immunotherapy requires a multi-disciplinary approach, taking the overall clinical context into consideration.

Furthermore, a trend towards a combination of neoadjuvant and adjuvant immunotherapeutic application can be observed; while participants in earlier trials such as the CheckMate 816 [5] mostly underwent a neoadjuvant regimen of immunotherapy, more recent studies such as the RATIONALE-315 [17], Neotorch [18], or the CheckMate 77T [19] have expanded the use of immunotherapy to a perioperative setting.

The retrospective cohort study of Shuai et al. [24] focused on elderly patients (≥65 years), and their findings suggest higher survival and pathological response rates in those patients treated with immunochemotherapy. However, grade ≥ 3 TRAEs were more likely to occur in the intervention group. RCTs such as the CheckMate 816 study [5,34] provided subgroup analyses, also comparing outcomes between patients under the age of 65 and patients with 65 years and older. The data indicated that elderly patients undergoing immunochemotherapy achieved superior outcomes compared to elderly patients in the control group, though the magnitude of benefit was slightly lower compared to patients under the age of 65 receiving immunochemotherapy. However, as the RCTs included in this review did not primarily focus on the effect of immunochemotherapy in the elderly population, future RCTs with this specific primary endpoint are needed for further conclusions.

The findings of this study align with the results of previously conducted systematic reviews:

The study of Li et al. [38] showed that the combination of neoadjuvant immunotherapy with chemotherapy results in higher survival rates yet also in a slightly increased risk of TRAEs.

According to Sorin et al. [39], patients treated with neoadjuvant chemoimmunotherapy experienced improved survival and higher pathological response rates compared to the control group. The authors concluded no increased relative risk of TRAEs in their review.

Zhang et al. [40] reported a survival benefit in the group receiving neoadjuvant or perioperative immunochemotherapy, as well as a higher incidence of adverse events in the case of long-term treatment.

There are certain limitations that should be taken into consideration when evaluating the findings of this review: the literature search was limited to studies published in English or German and there is a possibility of publication bias. Another limitation to consider is the inclusion of prospective and retrospective cohort studies: the non-randomized nature of these studies makes them susceptible to selection bias and confounding, potentially reducing the certainty and strength of evidence provided. Also, besides meeting the eligibility criteria of this review, the included studies differed in terms of study design, population, intervention, and outcome characteristics, as well as in the endpoints of interest and mechanism of conduct. Sample sizes varied considerably across the individual studies and study designs ranged from single-center to multicenter, multinational studies. The disease stages of participants varied across the individual studies and the immunotherapeutic regimens differed as various types of agents were used, with some studies focusing on one immune checkpoint inhibitor and others analyzing outcomes of four to seven different agents. Another limitation of this review relates to the variability in extent and measures of reported outcomes and their statistical significance, the immaturity of overall survival data, and heterogenous PD-L1 assessment criteria. This heterogeneity in outcome data limits the comparability of the individual studies and represents a weakness of this review. An additional aspect to consider is that, for the study by Lei et al. (2023) [22], the median DFS and EFS were not yet reached in either arm and, instead, estimates were provided. The absence of mature survival data also limits direct comparability and reduces the certainty of the respective data. Moreover, the reporting of TRAEs was marked by heterogeneity with varying extent of data availability. Also, while most studies explicitly reported assessing TRAEs according to the CTCAE, some did not specify the grading system applied. In the context of TRAEs, another limitation is that only some studies provided information on the proportion of immune-mediated adverse events.

The results of this review are consistent with the improved clinical outcomes in chemoimmunotherapy-treated patients reported by previous studies. While these findings promote the expanding role of immunotherapy in clinical practice, the trend towards a higher frequency and severity of TRAEs associated with this treatment approach should be considered. The current lack and inconsistency of data on TRAEs emphasize the need for future research on this particular endpoint to draw clear conclusions and to subsequently adjust clinical practice especially in terms of patient selection and management of TRAEs.

## 5. Conclusions

The combination of chemotherapy with immunotherapy results in superior survival outcomes and higher pathological response rates in patients with resectable NSCLC. Findings on TRAEs are marked by heterogeneity and the extent of available data varies across the individual studies. However, data indicate a higher frequency and severity of TRAEs in patients treated with immunochemotherapy, yet those adverse events were mostly tolerable and manageable. Further studies and standardized reporting on TRAEs are needed to draw definite conclusions and to subsequently guide clinical practice.

## Figures and Tables

**Table 1:** Risk of bias assessment of randomized controlled trials [12,13].

Year of Publication	Authors (Study Name)	Domain 1	Domain 2	Domain 3	Domain 4	Domain 5	Overall Risk of Bias
2025	Yue et al. [17]	Low risk	Low risk	Low risk	Low risk	Low risk	Low risk
2024	Lu et al. [18]	Low risk	Some concerns	Some concerns	Low risk	Low risk	Some concerns
2024	Cascone et al. [19]	Low risk	Low risk	Low risk	Low risk	Low risk	Low risk
2023	Wakelee et al. [6]	Low risk	Low risk	Low risk	Low risk	Low risk	Low risk
2023	Heymach et al. [20]	Low risk	Low risk	Low risk	Low risk	Low risk	Low risk
2023	Provencio et al. [21]	Low risk	Some concerns	Some concerns	Low risk	Low risk	Some concerns
2023	Lei, J. et al. [22]	Low risk	Low risk	Low risk	Some concerns	Low risk	Some concerns
2022	Forde et al. [5]	Low risk	Some concerns	Low risk	Low risk	Low risk	Some concerns

**Table 2:** Risk of bias assessment of prospective and retrospective cohort studies [14,15].

Year of Publication	Authors (Study Name)	Selection	Comparability	Outcome	Overall
2025	Lei, M. et al. [23]	3/4	2/2	2/3	7/9
2025	Shuai et al. [24]	4/4	2/2	3/3	9/9
2025	Wang, Y. et al. [25]	3/4	2/2	2/3	7/9
2024	Wang, K. et al. [26]	4/4	2/2	3/3	9/9
2024	Yang, Z. et al. [27]	4/4	2/2	2/3	8/9
2023	Zhou et al. [28]	4/4	0/2	2/3	6/9
2022	Liu et al. [29]	4/4	2/2	2/3	8/9
2018	Yang, C.-F.J. et al. [30]	4/4	0/2	3/3	7/9

**Table 3:** Study and population characteristics.

Author (Trial Name), Year	Country	Study Design	Total Sample Size: *n* (Intervention vs. Comparison)	Disease Stage	Median Age (Range)/Age (Intervention vs. Comparison)	Sex: *n* (%) (Intervention vs. Comparison)	Reference Number
Yue et al. (RATIONALE-315), 2025 [17]	China	Phase III RCT (double-blinded), multicenter	453 (226 vs. 227)	IIA-IIIA	62 (57–67) vs. 63 (56–68)	Male: 205 (91%) vs. Male: 205 (90%)	[17,31]
Lei, M. et al., 2025 [23]	China	Retrospective cohort study, single-center	72 (24 vs. 48)	IIA-IIIB (PLELC)	52.5 (29–71) vs. 50.5 (29–66)	Male: 10 (41.7%) vs. Male: 21 (43.8%)	[23]
Shuai et al., 2025 [24]	China	Retrospective cohort study, single-center	140 (93 vs. 47)	IB-IIIB	70 (67.0–73.5) vs. 70 (66.3–71.0)	Male: 83 (89.2%) vs. Male: 38 (80.9%)	[24]
Wang, Y. et al., 2025 [25]	China	Retrospective cohort study, single-center	71 (46 vs. 25)	IIIA-IIIC	58.5 (52.0–64.0) vs. 59 (54.5–64.5)	Male: 38 (82.6%) vs. Male: 20 (80.0%)	[25]
Lu et al. (Neotorch), 2024 [18]	China	Phase III RCT (double-blinded), multicenter	404 (202 vs. 202)	IIIA-IIIB	62 (56–65) vs. 61 (56–65)	Male: 181 (89.6%) vs. Male: 189 (93.6%)	[18]
Cascone et al. (CheckMate 77T), 2024 [19]	Multinational	Phase III RCT (double-blinded), multicenter	461 (229 vs. 232)	IIA-IIIB	66 (37–83) vs. 66 (35–86)	Male: 167 (72.9%) vs. Male: 160 (69.0%)	[19,32]
Wang, K. et al., 2024 [26]	China	Prospective cohort study, multicenter	84 (42 vs. 42) Final analysis: 81 (41 vs. 40)	IIA-IIIA	65 (60–70) vs. 65 (59–67)	Male: 33 (80.5%) vs. Male: 27 (67.5%)	[26]
Yang, Z. et al. (NeoR-World), 2024 [27]	China	Retrospective cohort study, multicenter	540 (270 vs. 270)	IA-III	61 (56–66) vs. 59 (53–64) (Including patients with EGFR/ALK mutations)	Male: 369 (90.4%) vs. Male: 551 (80.6%) (Including patients with EGFR/ALK mutations)	[27]
Wakelee et al. (KEYNOTE-671), 2023 [6]	Multinational	Phase III RCT (double-blinded), multicenter	797 (397 vs. 400)	IIA-IIIB	63 (26–83) vs. 64 (35–81)	Male: 279 (70.3%) vs. Male: 284 (71.0%)	[6,33]
Heymach et al. (AEGEAN), 2023 [20]	Multinational	Phase III RCT (double-blinded), multicenter	802 (400 vs. 402)	IIA-IIIB	65 (30–88) vs. 65 (39–85)	Male: 252 (68.9%) vs. Male: 278 (74.3%)	[20]
Provencio et al. (NADIM II), 2023 [21]	Spain	Phase II RCT (open-label), multicenter	86 (57 vs. 29)	IIIA-IIIB	65 (58–70) vs. 63 (57–66)	Male: 36 (63%) vs. Male: 16 (55%)	[21]
Zhou et al., 2023 [28]	China	Retrospective cohort study, single-center	59 (26 vs. 33)	IIIA-IIIB	≤60 years: 61.6% >60 years: 38.4% vs. ≤60 years: 48.4% >60 years: 51.6%	Male: 24 (92.4%) vs. Male: 27 (81.9%)	[28]
Lei, J. et al. (TD-FOREKNOW), 2023 [22]	China	Phase II RCT (open-label), multicenter	94 (47 vs. 47) Final analysis: 88 (43 vs. 45)	IIIA-IIIB	61 (54–65) vs. 61 (54–65)	Male: 34 (79.1%) vs. Male: 40 (88.9%)	[22]
Forde et al. (CheckMate 816), 2022 [5]	Multinational	Phase III RCT (open-label), multicenter	358 (179 vs. 179)	IIIA-IB	64 (41–82) vs. 65 (34–84)	Male: 128 (71.5%) vs. Male: 127 (70.9%)	[5,34]
Liu et al., 2022 [29]	China	Retrospective cohort study, single-center	170 (79 vs. 91)	IB-IIIB	≥60 years: 64.6% <60 years: 35.4% vs. ≥60 years: 53.8% <60 years: 46.2%	Male: 66 (83.5%) vs. Male: 75 (82.4%)	[29]
Yang, C.-F.J. et al., 2018 [30]	United States	Prospective and retrospective cohort study, single-center	55 (13 vs. 42)	IB-IV	59 (51–75) vs. 62 (33–76)	Male: 5 (38%) vs. Male: 21 (50%)	[30]

RCT: randomized controlled trial; PLELC: pulmonary lymphoepithelioma-like carcinoma; EGFR: epidermal growth factor receptor; ALK: anaplastic lymphoma kinase.

**Table 4:** Intervention and comparison characteristics.

Study	Intervention (*n*)	Immunotherapy Timing	Comparison (*n*)
Yue et al. (RATIONALE-315), 2025 [17]	Tislelizumab + Cisplatin/Carboplatin + Paclitaxel/Pemetrexed	Perioperative	Cisplatin/Carboplatin + Paclitaxel/Pemetrexed
Lei, M. et al., 2025 [23]	Tislelizumab (9) Sintilimab (7) Toripalimab (4) Pembrolizumab (3) Camrelizumab (1) + Platinum + Taxanes (20) Platinum + Gemcitabine (2) Paclitaxel (2)	Perioperative	Platinum + Taxanes (31) Platinum + Gemcitabine (11) Platinum + Pemetrexed (6)
Shuai et al., 2025 [24]	Camrelizumab (30) Tislelizumab (20) Pembrolizumab (16) Nivolumab (14) Sintilimab (11) Durvalumab (2) + Cisplatin/Carboplatin + Paclitaxel	Neoadjuvant	Cisplatin/Carboplatin + Paclitaxel
Wang, Y. et al., 2025 [25]	NR + Platinum-based therapy	Perioperative	Platinum-based therapy
Lu et al. (Neotorch), 2024 [18]	Toripalimab + Cisplatin/Carboplatin + Docetaxel/Paclitaxel/Pemetrexed	Perioperative	Cisplatin/Carboplatin + Docetaxel/Paclitaxel/Pemetrexed
Cascone et al. (CheckMate 77T), 2024 [19]	Nivolumab + Cisplatin/Carboplatin	Perioperative	Cisplatin/Carboplatin
Wang, K. et al., 2024 [26]	PD-1 monoclonal antibody + Cisplatin + Paclitaxel/Pemetrexed	Neoadjuvant	Cisplatin + Paclitaxel/Pemetrexed
Yang, Z. et al. (NeoR-World), 2024 [27]	Pembrolizumab/Camrelizumab/Tislelizumab/Sintilimab/Nivolumab/Durvalumab/Toripalimab/(Sintilimab + Nivolumab) + Cisplatin/Carboplatin/Nedaplatin/Oxaliplatin/Loplatin + Paclitaxel/Gemcitabine/Pemetrexed	Perioperative	Platinum-based therapy
Wakelee et al. (KEYNOTE-671), 2023 [6]	Pembrolizumab + Cisplatin + Gemcitabine/Pemetrexed	Perioperative	Cisplatin + Gemcitabine/Pemetrexed
Heymach et al. (AEGEAN), 2023 [20]	Durvalumab + Cisplatin/Carboplatin	Perioperative	Cisplatin/Carboplatin
Provencio et al. (NADIM II), 2023 [21]	Nivolumab + Carboplatin + Paclitaxel	Perioperative	Carboplatin + Paclitaxel
Zhou et al., 2023 [28]	Pembrolizumab/Tislelizumab/Sintilimab/Camrelizumab/Nivolumab + Platinum-based therapy (+ Gemcitabine/Etoposide)	Neoadjuvant	Platinum-based therapy
Lei, J. et al. (TD-FOREKNOW), 2023 [22]	Camrelizumab + Cisplatin/Carboplatin/Nedaplatin + Paclitaxel	Neoadjuvant	Cisplatin/Carboplatin/Nedaplatin + Paclitaxel
Forde et al. (CheckMate 816), 2022 [5]	Nivolumab + Cisplatin/Carboplatin	Neoadjuvant	Cisplatin/Carboplatin
Liu et al., 2022 [29]	Pembrolizumab (34) Nivolumab (20) Sintilimab (13) Camrelizumab (12) + Platinum + Paclitaxel (50) Platinum + Pemetrexed (26) Other regimens (3)	Neoadjuvant	Platinum + Paclitaxel: 52 Platinum + Pemetrexed: 32 Other regimens: 7
Yang, C.-F.J. et al., 2018 [30]	Ipilimumab + Cisplatin/Carboplatin + Paclitaxel	Neoadjuvant	Platinum-based therapy

NR: not reported; PD-1: programmed cell death protein 1.

**Table 5:** Survival outcomes.

Year	Authors (Study Name)	Survival (Intervention vs. Comparison)	HR (95% CI); *p*-Value
2025	Yue et al. (RATIONALE-315) [17]	12 mo EFS: 80% vs. 68%	0.56 (0.40–0.79); *p* = 0.0003
		24 mo EFS: 68% vs. 52%	0.56 (0.40–0.79); *p* = 0.0003
		36 mo EFS 64.7% vs. 48.0%	0.58 (0.43–0.79); NR
		12 mo OS: 95% vs. 91%	0.62 (0.39–0.98); *p* = 0.019
		24 mo OS: 89% vs. 79%	0.62 (0.39–0.98); *p* = 0.019
		36 mo OS: 79.3% vs. 69.3%	0.65 (0.45–0.93); 0.009
2025	Lei, M. et al. [23]	Median EFS: NE vs. 35.0 mo	0.42 (0.19–0.93); *p* = 0.031
		Median OS: NE vs. NE	0.27 (0.04–1.91); *p* = 0.188
		Survival rates at median of 47.0 mo (2.0–135.0 mo): 100% vs. 79.2%	NR
2025	Shuai et al. [24]	Median DFS: NE vs. 19.4 mo	0.247 (0.122–0.501); *p* < 0.001
		2 yr DFS rate: 83.1% vs. 45.2%	NR
		Median OS: NE vs. 41.8 mo	0.265 (0.115–0.611); *p* < 0.001
		2 yr OS rate: 89.8% vs. 74.2%	NR
2025	Wang, Y. et al. [25]	Median DFS: NE vs. 15 mo	0.186 (0.073–0.479); *p* < 0.001
		2 yr DFS rate: 85.0% vs. 36.3%	NR
		2 yr OS rate: 85.1% vs. 82.5%	NR
2024	Lu et al. (Neotorch) [18]	Median DFS: NE vs. 22.0 mo	0.49 (0.31–0.76); *p* < 0.001
		Median EFS: NE vs. 15.5 mo	0.40 (0.27–0.57); *p* < 0.001
		Median OS: NE vs. 30.4 mo	0.62 (0.38–1.00); *p* = 0.05
2024	Cascone et al. (CheckMate 77T) [19]	Median EFS: 46.6 mo vs. 16.9 mo	0.61 (0.46–0.80); NR
		18 mo EFS: 70.2% vs. 50.0%	0.58 (97.36% CI: 0.42–0.81); *p* < 0.001
		30 mo EFS: 61% vs. 43%	0.61 (0.46–0.80); NR
		30 mo OS: 78% vs. 72%	0.85 (0.58–1.25); NR
2024	Wang, K. et al. [26]	NR	NR
2024	Yang, Z. et al. (NeoR-World) [27]	2 yr DFS: 80.5% vs. 63.6%	0.50 (0.36–0.70); *p* < 0.001
		2 yr OS: 91.6% vs. 83.4%	0.91 (0.64–1.30); *p* = 0.604
2023	Wakelee et al. (KEYNOTE-671) [6]	Median EFS: 47.2 mo vs. 18.3 mo	0.59 (0.48–0.72); NR
		24 mo EFS: 62.4% vs. 40.6%	0.58 (0.46–0.72); *p* < 0.001
		36 mo EFS: 54% vs. 35%	0.59 (0.48–0.72); NR
		24 mo OS: 80.9% vs. 77.6%	NR; *p* = 0.02
		36 mo OS: 71% vs. 64%	0.72 (0.56–0.93); *p* = 0.0052 (one-sided) vs. *p* = 0.0054 (one-sided)
2023	Heymach et al. (AEGEAN) [20]	Median EFS: NE vs. 25.9 mo	0.68 (0.53–0.88); *p* = 0.004
		12 mo EFS: 73.4% vs. 64.5%	NR
		24 mo EFS: 63.3% vs. 52.4%	NR
2023	Provencio et al. (NADIM II) [21]	24 mo PFS: 67.2% vs. 40.9%	0.47 (0.25–0.88); NR
		24 mo OS: 85.0% vs. 63.6%	0.43 (0.19–0.98); NR
2023	Zhou et al. [28]	2 yr DFS rate: 76.9% vs. 63.8%	NR; *p* = 0.129
2023	Lei, J. et al. (TD-FOREKNOW) [22]	Estimated 12 mo DFS: 93.2% vs. 81.4% Estimated 24 mo DFS: 78.4% vs. 71.7%	0.54 (0.19–1.54); NR
		Estimated 12 mo EFS: 93.0% vs. 76.9% Estimated 24 mo EFS: 76.9% vs. 67.6%	0.52 (0.21–1.29); NR
2022	Forde et al. (CheckMate 816) [5]	Median EFS: 31.6 mo vs. 20.8 mo	0.63 (97.38% CI: 0.43–0.91); *p* = 0.005
		4 yr EFS rate: 49% vs. 38%	0.66 (0.49–0.90); NR
		4 yr OS rate: 71% vs. 58%	0.71 (98.36% CI: 0.47–1.07); *p* = 0.0451
		Median OS: NE vs. NE	0.57 (99.67% CI: 0.30–1.07); *p* = 0.008
2022	Liu et al. [29]	2 yr DFS: 67.2% vs. 39.5%	NR; *p* = 0.019
2018	Yang, C.-F.J. et al. [30]	30-day survival: 100% vs. 100%	NR
		90-day survival: 100% vs. 98%	NR

HR: hazard ratio; CI: confidence interval; mo: month(s); EFS: event-free survival; NR: not reported; OS: overall survival; NE: not estimable; DFS: disease-free survival; yr: year; PFS: progression-free survival.

**Table 6:** Pathological response outcomes.

Year	Authors (Study Name)	pCR (Intervention vs. Comparison)	MPR (Intervention vs. Comparison)	pCR: (95% CI); *p*-Value	MPR: (95% CI); *p*-Value
2025	Yue et al. (RATIONALE-315) [17]	pCR: 41% vs. 6%	MPR: 56% vs. 15%	RD: 35% (28–42); *p* < 0.0001	RD: 41% (33–49); *p* < 0.0001
2025	Lei, M. et al. [23]	pCR: 33.3% vs. 4.2%	MPR: 54.2% vs. 12.5%	OR: 1.44 (1.08–1.92); *p* < 0.002	OR: 1.91 (1.22–2.99); *p* < 0.001
2025	Shuai et al. [24]	pCR: 32.2% vs. 16.1%	MPR: 64.4% vs. 25.8%	NR; *p* = 0.101	NR; *p* < 0.001
2025	Wang, Y. et al. [25]	pCR: 36.4% vs. 8.3%	MPR: 65.9% vs. 16.7%	OR: 7.215 (1.436–36.256); *p* = 0.016	OR: 11.442 (2.982–43.898; *p* < 0.001
2024	Lu et al. (Neotorch) [18]	pCR: 24.8% vs. 1.0%	MPR: 48.5% vs. 8.4%	RD: 23.7% (17.6–29.8); *p* < 0.001	RD: 40.2% (32.2–48.1); *p* < 0.001
2024	Cascone et al. (CheckMate 77T) [19]	pCR: 25.3% vs. 4.7%	MPR: 35.4% vs. 12.1%	OR: 6.64 (3.40–12.97); NR	OR: 4.01 (2.48–6.49); NR
2024	Wang, K. et al. [26]	pCR: 24.39% vs. 5%	NR	NR; *p* = 0.026	NR
2024	Yang, Z. et al. (NeoR-World) [27]	pCR: 36.3% vs. 7.4%	MPR: 61.1% vs. 23.0%	OR: 7.12 (4.33–12.3); *p* < 0.001	OR: 5.27 (3.64–7.71); *p* < 0.001
2023	Wakelee et al. (KEYNOTE-671) [6]	pCR: 18.1% vs. 4.0%	MPR: 30.2% vs. 11.0%	RD: 14.2% (10.1–18.7); *p* < 0.0001	RD: 19.2% (13.9–24.7); *p* < 0.0001
2023	Heymach et al. (AEGEAN) [20]	pCR: 17.2% vs. 4.3%	MPR: 33.3% vs. 12.3%	RD: 13.0% (8.7–17.6); *p* < 0.001	RD: 21.0% (15.1–26.9); *p* < 0.001
2023	Provencio et al. (NADIM II) [21]	pCR: 37.0% vs. 7.0%	MPR: 53.0% vs. 14.0%	RR: 5.34 (1.34–21.23); *p* = 0.02	RR: 3.82 (1.49–9.79); NR
2023	Zhou et al. [28]	pCR: 34.6% vs. 3.0%	MPR: 65.3% vs. 15.1%	RR: 11.423 (1.544–84.493); *p* = 0.004	RR: 4.315 (1.836–10.142); *p* < 0.001
2023	Lei, J. et al. (TD-FOREKNOW) [22]	pCR: 32.6% vs. 8.9%	MPR: 65.1% vs. 15.6%	NR; *p* = 0.008	NR; *p* < 0.001
2022	Forde et al. (CheckMate 816) [5]	pCR: 24.0% vs. 2.2%	MPR: 36.9% vs. 8.9%	OR: 13.94 (99% CI: 3.49–55.75); *p* < 0.001	OR: 5.70 (3.16–10.26); NR
2022	Liu et al. [29]	pCR + MPR: 53.2% vs. 14.3%	pCR + MPR: 53.2% vs. 14.3%	NR; *p* < 0.001	NR; *p* < 0.001
2018	Yang, C.-F.J. et al. [30]	pCR: 15% vs. 12%	NR	NR; *p* = 0.66	NR

pCR: pathological complete response; MPR: major pathological response; CI: confidence interval; RD: risk difference; OR: odds ratio; NR: not reported; RR: relative risk.

**Table 7:** Adverse events and safety outcomes.

Year	Authors (Study Name)	TRAEs Any Grade (%) (Intervention vs. Comparison)	Grade ≥ 3 TRAEs (%) (Intervention vs. Comparison)	Serious TRAEs (%) (Intervention vs. Comparison)	Discontinuation Due to TRAEs (%) (Intervention vs. Comparison)	Fatal TRAEs (%) (Intervention vs. Comparison)
2025	Yue et al. (RATIONALE-315) [17]	99% vs. >99%	73.0% vs. 67.3%	15% vs. 8%	13% vs. 9%	2% vs. 1%
2025	Lei, M. et al. [23]	91.7% vs. 89.6%	41.7% vs. 39.6%	NR	NR	NR
2025	Shuai et al. [24]	NR	19.4% vs. 8.5%	NR	NR	NR
2025	Wang, Y. et al. [25]	80.4% vs. 64.0%	10.9% vs. 8.0%	NR	NR	0% vs. 0%
2024	Lu et al. (Neotorch) [18]	99.5% vs. 98.5%	63.4% vs. 54.0%	NR	9.4% vs. 7.4%	3.0% vs. 2.0%
2024	Cascone et al. (CheckMate 77T) [19]	97.4% vs. 97.8% (Any Cause) 89.0% vs. 87.0% (possibly related)	32.5% vs. 25.2%	19.3% vs. 9.6%	19.3% vs. 7.4%	0.9% vs. 0%
2024	Wang, K. et al. [26]	NR	NR	NR	NR	NR
2024	Yang, Z. et al. (NeoR-World) [27]	NR	NR	NR	NR	NR
2023	Wakelee et al. (KEYNOTE-671) [6]	96.7% vs. 95.0%	44.9% vs. 37.3%	17.7% vs. 14.3%	12.6% vs. 5.3%	1.0% vs. 0.8%
2023	Heymach et al. (AEGEAN) [20]	96.5% vs. 94.7% (Any Cause) 86.8% vs. 80.7% (possibly related)	42.4% vs. 43.2% (Any Cause) 32.4% vs. 32.9% (possibly related)	37.7% vs. 31.4%	12.0% vs. 6.0%	1.7% vs. 0.5%
2023	Provencio et al. (NADIM II) [21]	88% vs. 90%	19% vs. 10%	NR	7.0% vs. 3.4%	NR
2023	Zhou et al. [28]	84.6% vs. 63.6%	4.6% vs. 33.3%	NR	NR	NR
2023	Lei, J. et al. (TD-FOREKNOW) [22]	95.3% vs. 88.9%	25.6% vs. 11.1%	NR	NR	0% vs. 0%
2022	Forde et al. (CheckMate 816) [5]	82.4% vs. 88.6%	33.5% vs. 36.9%	11.9% vs. 10.2%	10.2% vs. 9.7%	0% vs. 1.7%
2022	Liu et al. [29]	NR	NR	NR	NR	NR
2018	Yang, C.-F.J. et al. [30]	NR	NR	NR	NR	NR

TRAEs: treatment-related adverse events; NR: not reported.

## Data Availability

No new data were created or analyzed in this study. Data sharing is not applicable to this article.

## Supplementary Materials

The following supporting information can be downloaded at: https://www.mdpi.com/article/10.3390/cancers18122002/s1, Table S1: PRIMSA Abstract Checklist; Table S2: PRISMA 2020 Main Checklist. Reference [16] is cited in the Supplementary Materials.

## Abbreviations

The following abbreviations are used in this manuscript:

ALK	Anaplastic Lymphoma Kinase
CTCAE	Common Terminology Criteria for Adverse Events
DFS	Disease-Free Survival
EFS	Event-Free Survival
EGFR	Epidermal Growth Factor Receptor
ICD	Immunogenic Cell Death
ICTRP	International Clinical Trials Registry Platform
MPR	Major Pathological Response
NK	Natural Killer
NSCLC	Non-Small-Cell Lung Cancer
OS	Overall Survival
pCR	Pathological Complete Response
PD-1	Programmed Cell Death Protein 1
PD-L1	Programmed Death-Ligand 1
PFS	Progression-Free Survival
PLELC	Pulmonary Lymphoepithelioma-Like Carcinoma
RCT	Randomized Controlled Trial
TRAEs	Treatment-Related Adverse Events
WHO	World Health Organization

## Appendix A

**Table A1:** PubMed search strategy.

Date	Results	Search Details	Query	Search Number
2025/07/23	159	(“Non-Small Cell Lung Cancer”[Title/Abstract] OR “NSCLC”[Title/Abstract] OR “carcinoma, non small cell lung”[MeSH Terms]) AND (“resect*”[Title/Abstract] OR “surg*”[Title/Abstract] OR “pulmonary surgical procedures”[MeSH Terms]) AND (“immunotherap*”[Title/Abstract] OR (“Immune”[Title/Abstract] AND “Checkpoint”[Title/Abstract] AND “inhibit*”[Title/Abstract]) OR (“Pembrolizumab”[Title/Abstract] OR “Nivolumab”[Title/Abstract] OR “Atezolizumab”[Title/Abstract] OR “Durvalumab”[Title/Abstract] OR “Ipilimumab”[Title/Abstract]) OR “immunotherapy”[MeSH Terms] OR “immune checkpoint inhibitors”[MeSH Terms]) AND (“chemotherap*”[Title/Abstract] OR “anti neoplastic”[Title/Abstract] OR “antineoplastic agents”[MeSH Terms] OR “drug therapy, combination”[MeSH Terms]) AND (“Perioperative”[Title/Abstract] OR “Adjuvant”[Title/Abstract] OR “Neoadjuvant”[Title/Abstract] OR “Preoperative”[Title/Abstract] OR “Postoperative”[Title/Abstract]) AND (“english”[Language] OR “german”[Language]) AND 2015/01/01:3000/12/31[Date—Publication] AND “humans”[MeSH Terms] AND (“randomized controlled trial”[Publication Type] OR (“Randomized”[Title/Abstract] AND “Controlled”[Title/Abstract] AND “Trial”[Title/Abstract]) OR “Randomised Controlled Trial”[Title/Abstract] OR (“systematic review”[Publication Type] OR (“Systematic”[Title/Abstract] AND “Review”[Title/Abstract]) OR (“meta analysis”[Publication Type] OR “meta analysis”[Title/Abstract] OR “meta analysis”[Title/Abstract])) OR (“Cohort Study”[Title/Abstract] OR “Cohort”[Title/Abstract] OR “Prospective Cohort”[Title/Abstract] OR “Retrospective Cohort”[Title/Abstract] OR “Prospective Study”[Title/Abstract] OR “Retrospective Study”[Title/Abstract]))	(#1 OR #2) AND (#3 OR #4) AND (#5 OR #6 OR #7 OR #8 OR #9) AND (#10 OR #11 OR #12) AND #13 AND #14 AND #15 AND #16 AND (#17 OR #18 OR #19)	20
2025/07/23	1,364,693	“Cohort Study”[Title/Abstract] OR “Cohort”[Title/Abstract] OR “Prospective Cohort”[Title/Abstract] OR “Retrospective Cohort”[Title/Abstract] OR “Prospective Study”[Title/Abstract] OR “Retrospective Study”[Title/Abstract]	(((((„Cohort Study”[Title/Abstract]) OR (Cohort[Title/Abstract])) OR („Prospective Cohort”[Title/Abstract])) OR („Retrospective Cohort”[Title/Abstract])) OR („Prospective Study”[Title/Abstract])) OR („Retrospective Study”[Title/Abstract])	19
2025/07/23	589,104	“systematic review”[Publication Type] OR (“Systematic”[Title/Abstract] AND “Review”[Title/Abstract]) OR (“meta analysis”[Publication Type] OR “meta analysis”[Title/Abstract] OR “meta analysis”[Title/Abstract])	Systematic Review[Publication Type] OR (Systematic[Title/Abstract] AND Review[Title/Abstract]) OR (Meta-Analysis[Publication Type] OR Meta- Analysis[Title/Abstract] OR „Meta Analysis”[Title/Abstract])	18
2025/07/23	746,089	“randomized controlled trial”[Publication Type] OR (“Randomized”[Title/Abstract] AND “Controlled”[Title/Abstract] AND “Trial”[Title/Abstract]) OR “Randomised Controlled Trial”[Title/Abstract]	Randomized Controlled Trial[Publication Type] OR (Randomized[Title/Abstract] AND Controlled[Title/Abstract] AND Trial[Title/Abstract]) OR „Randomised Controlled Trial” [Title/Abstract]	17
2025/07/23	22,840,636	“humans”[MeSH Terms]	“humans”[Filter]	16
2025/07/23	14,429,963	2015/01/01:3000/12/31[Date—Publication]	(“2015”[Date—Publication]: “3000”[Date—Publication])	15
2025/07/23	35,157,946	“english”[Language] OR “german”[Language]	(“english”[Language]) OR (“german”[Language])	14
2025/07/23	1,217,367	“Perioperative”[Title/Abstract] OR “Adjuvant”[Title/Abstract] OR “Neoadjuvant”[Title/Abstract] OR “Preoperative”[Title/Abstract] OR “Postoperative”[Title/Abstract]	((((Perioperative[Title/Abstract]) OR (Adjuvant[Title/Abstract])) OR (Neoadjuvant[Title/Abstract])) OR (Preoperative[Title/Abstract])) OR (Postoperative[Title/Abstract])	13
2025/07/23	370,851	“drug therapy, combination”[MeSH Terms]	Drug Therapy, Combination[MeSH Terms]	12
2025/07/23	525,843	“antineoplastic agents”[MeSH Terms]	Antineoplastic Agents[MeSH Terms]	11
2025/07/23	563,184	“chemotherap*”[Title/Abstract] OR “anti neoplastic”[Title/Abstract]	(Chemotherap*[Title/Abstract]) OR (Anti- neoplastic[Title/Abstract])	10
2025/07/23	15,782	“immune checkpoint inhibitors”[MeSH Terms]	Immune Checkpoint Inhibitors[MeSH Terms]	9
2025/07/23	364,114	“immunotherapy”[MeSH Terms]	Immunotherapy[MeSH Terms]	8
2025/07/23	25,540	“Pembrolizumab”[Title/Abstract] OR “Nivolumab”[Title/Abstract] OR “Atezolizumab”[Title/Abstract] OR “Durvalumab”[Title/Abstract] OR “Ipilimumab”[Title/Abstract]	((((Pembrolizumab[Title/Abstract]) OR (Nivolumab[Title/Abstract])) OR (Atezolizumab[Title/Abstract])) OR (Durvalumab[Title/Abstract])) OR (Ipilimumab[Title/Abstract])	7
2025/07/23	47,993	“Immune”[Title/Abstract] AND “Checkpoint”[Title/Abstract] AND “inhibit*”[Title/Abstract]	((Immune[Title/Abstract]) AND (Checkpoint[Title/Abstract])) AND (Inhibit*[Title/Abstract])	6
2025/07/23	185,864	“immunotherap*”[Title/Abstract]	Immunotherap*[Title/Abstract]	5
2025/07/23	83,533	“pulmonary surgical procedures”[MeSH Terms]	Pulmonary Surgical Procedures[MeSH Terms]	4
2025/07/23	2,829,407	“resect*”[Title/Abstract] OR “surg*”[Title/Abstract]	(Resect*[Title/Abstract]) OR (Surg*[Title/Abstract])	3
2025/07/23	79,655	“carcinoma, non small cell lung”[MeSH Terms]	Carcinoma, Non-Small-Cell Lung[MeSH Terms]	2
2025/07/23	101,255	“Non-Small Cell Lung Cancer”[Title/Abstract] OR “NSCLC”[Title/Abstract]	(„Non-Small Cell Lung Cancer”[Title/Abstract]) OR (NSCLC[Title/Abstract])	1
